# Exploring the role of cathepsin in rheumatoid arthritis

**DOI:** 10.1016/j.sjbs.2021.09.014

**Published:** 2021-09-13

**Authors:** Tapan Behl, Swati Chadha, Aayush Sehgal, Sukhbir Singh, Neelam Sharma, Rajwinder Kaur, Saurabh Bhatia, Ahmed Al-Harrasi, Sridevi Chigurupati, Ahmed Alhowail, Simona Bungau

**Affiliations:** a Chitkara College of Pharmacy, Chitkara University, Punjab, India; b Natural & Medical Sciences Research Centre, University of Nizwa, Nizwa, Oman; c Adjunct Professor, Amity Institute of Pharmacy, Amity University, Haryana, India; d Department of Medicinal Chemistry and Pharmacognosy, College of Pharmacy, Qassim University, Buraidah, Saudi Arabia; e Department of Pharmacology and Toxicology, College of Pharmacy, Qassim University, Buraidah, Saudi Arabia; f Department of Pharmacy, Faculty of Medicine and Pharmacy, University of Oradea, Oradea, Romania

**Keywords:** Cathepsin, Inflammation, Rheumatoid arthritis, Cysteine protease, Matrix-metalloprotease

## Abstract

Rheumatoid arthritis (RA) is a chronic inflammatory disease which is marked by leukocytes infiltration inside synovial tissue, joints and also inside synovial fluid which causes progressive destruction of joint cartilage. There are numerous genetical and lifestyle factors, responsible for rheumatoid arthritis. One such factor can be cysteine cathepsins, which act as proteolytic enzymes. These proteolytic enzyme gets activated at acidic pH and are found in lysosomes and are also termed as cysteine proteases. These proteases belong to papain family and have their elucidated role in musculoskeletal disorders. Numerous cathepsins have their targeted role in rheumatoid arthritis. These proteases are secreted through various cell types which includes matrix metalloproteases and papain like cysteine proteases. These proteases can potentially lead to bone and cartilage destruction which causes an immune response in case of inflammatory arthritis.

## 1 Introduction

Rheumatoid arthritis (RA), a chronic inflammatory disease is recognised as one of the most debilitating disorder characterised by infiltration of leukocytes inside bones and joints like synovial tissues, joints, and synovial fluid, leading to successive destruction of joint cartilage. The histological hallmarks of rheumatoid arthritis gets characterised via the development of aggressive pannus which invades inside bones and cartilage. The joint parenchymal lesions are detected with synovial hyperplasia and cellular infiltrate which contain numerous cells like monocytes/macrophages, lymphocytes as well as neutrophils. The abundantly present cells inside synovial fluid and tissues are macrophages/monocytes, exerting crucial role in pathogenesis of RA (Chadha et al., 2020, Vasiljeva et al., 2007, YASUDA et al., 2005, Hasilik et al., 2009). It leads to production of excessive amount of pro-inflammatory cytokines including tumor necrosis factor-a (TNF-a) and interleukin (IL-1), resulting in fibroblast hyper-proliferation, joint cartilage and bone destruction, and pannus formation. This pannus formation causes hyperplasia by attaching initially with synovial membrane and then degrading the cartilage. The degree of hyperplasia is co-related with the severity of cartilage erosions. The unremitting recruitment of macrophages and monocytes inside synovial tissue is carried out by via a process which gets controlled through chemotactic molecules and chemokines. An irreparable destruction of articular cartilage and bone is other hallmark of arthritis (Schett, 2008, Turk et al., 2012). The destruction of bone and cartilage occurs as a result of elevated levels of activated forms of the proteolytic enzymes. These proteolytic enzymes are responsible for the degradation of the cartilage aggrecan proteoglycan and bone collagen. MMPs are the widely investigated proteolytic enzymes as because of their action at neutral pH. However, an acidic pH allows cathepsins to act and degrade the cartilage. The major cysteine proteases which are involved in pathophysiology of joint destruction and rheumatoid arthritis comprises of cathepsin K and B. The exact and precise method of destruction in arthritis is not fully elucidated. Cysteine cathepsin family comprises of 11 members namely termed as cathepsins B, C, F, H, K, W, X, L, O, S, and Z. Cathepsin B, K, L, H and S are complicated and have their elaborated action in degradation of native collagen and in components of extracellular matrix. All cathepsin, except cathepsin K act as intracellular enzymes located inside lysosomes. The activity of cathepsins is entirely dependent on pH. They act at low pH i.e. below 7, is found in extracellular locations (such as resorption lacunae of osteoclast, around hip prosthesis), lysosomes (Martin, 2004, Riese et al., 1996). Previous studies have shown that cysteine cathepsin are ubiquitinous in nature and exert their action on skeletal tissues. It is believed that various cysteine cathepsins are involved in pathological condition like rheumatoid arthritis and osteoarthritis (Vizovišek et al., 2020, Arora et al., 2021). Numerous studies including molecular biologic technique, transgenic techniques were done on cathepsins in order to evaluate their role in rheumatoid arthritis. Numerous cathepsins which are known to have their role in rheumatoid arthritis includes cathepsin S, K, B and G. Enhanced activity of numerous proteases can be responsible for the destruction of articular cartilage as well as bone. These proteases are secreted through number of cell types which includes matrix metalloproteases and papain like cysteine proteases. These proteases can potentially lead to bone and cartilage destruction which causes an immune response in case of inflammatory arthritis. Studies have demonstrated about enhanced expression of cysteine cathepsin inside synovial fluid and synovial membrane. Although the complete mechanism is not elucidated yet and the studies conducted on them includes the in-vitro and in-vivo experimentation (Montague-Cardoso and Malcangio, 2020, Yue et al., 2020, Wang et al., 2020, Rowley et al., 2008). This review will be focusing on these cathepsins and their molecular role/expression in the pathogenesis of rheumatoid arthritis.

## 2 Spotlight on the role of cathepsins in rheumatoid arthritis

**Fig. 1:**
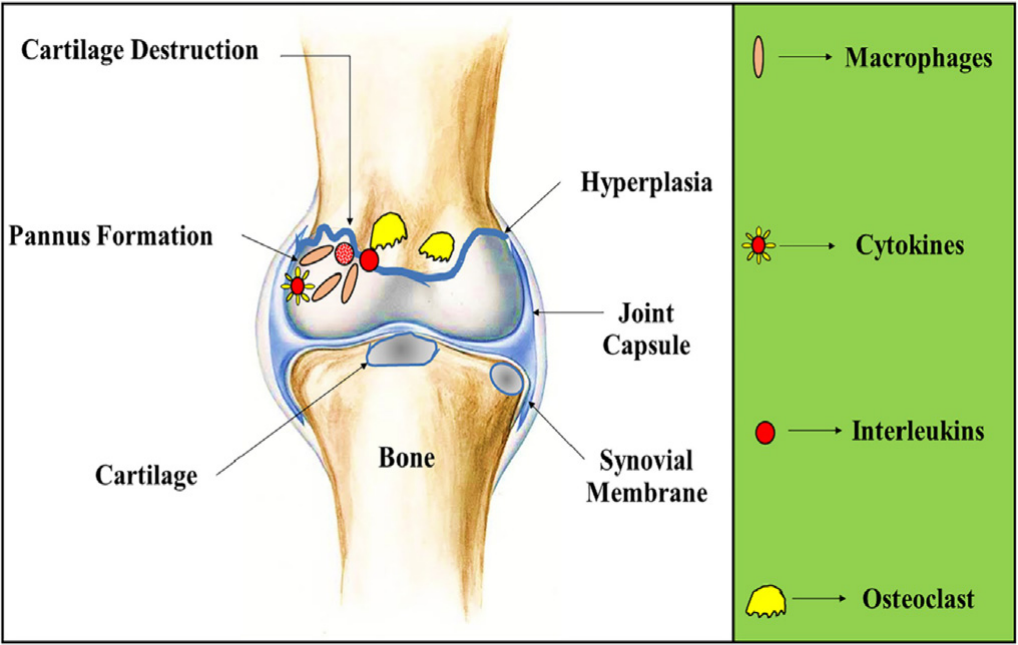
Process of accumulation of cytokines, osteoclast, interleukins and macrophages can lead to pannus formation which can further lead to cartilage destruction in patients with rheumatoid arthritis.

Rheumatoid arthritis is an auto-immune disorder. The complete mechanism by which it occurs is not fully elucidated but it is observed that in patients with RA, autoantibodies are produced against the components of extracellular matrix (ECM). This in turn causes destruction of immune system which in combination with synovial cells leading to disease progression. Diarthro-dial synovial joint comprises of particular connective tissues as well as a fibrous capsule. 70% of the material of bone is of inorganic nature (made of mineral compound known as hydroxyapatite), organic material denotes 20% (comprising of type I collage), and 10% contains water (Goto et al., 2003, Hou et al., 2002, Churigt et al., 2008). The bones can be classified into two types: porous trabecular bone (also termed as spongy bone) and dense cortical bone (termed as compact bone). The destruction of bones and type I collagen occurs as a result of osteoclasts which are bone-demineralizing as well as bone degrading cells. These are multinucleated cells which expresses calcitonin receptors, cathepsin K and tartrate-resistant phosphatase (TRAP). Osteoclast can acidify an area between bone matrix and cell membrane which is termed as resorption lacuna and process of bone destruction is shown via Fig. 1 (Fig. 1). The acidification of bone can lead to demineralization of bone as well as mineral components, causing liberalization of the matrix collagens (Ttinen et al., 2002, Ishida et al., 2021, Richard et al., 2021). This serves as an acidic media for cathepsin K in order to show its proteolytic activity, as a result of this bone resorption occurs. Bone mineralization is based on the secretion of acids, whose activity is dependent on concentration of proton pumps and carbonic anhydrase. Inside osteoclast, cathepsin B, L and K are detected (Richard et al., 2021, Yang et al., 2020). Cartilage comprises of liquid phase (water: about 60–85%) as well as solid phase (containing 15–20% type II collagen, 2–10% aggrecans). Synovial membrane serves as a tissue, soft in nature and is present between joint cavity and articular capsule. Synovium is the clear, lubricating and viscid fluid which is secreted via synovial fibroblast. Persistent inflammation of synovium/synovial fluid during arthritis can lead to membrane expansion via hyper-proliferation of fibroblast. These synovial fibroblast gets infiltrated with mononuclear cells (including B cells, T helper cells, and macrophages) which leads to the formation of invasive pannus tissue and enhanced protease expression (Gordon and Hahn, 2010, Neybecker et al., 2020, Trabandt et al., 1991, Cunnane et al., 1999).

**Fig. 2:**
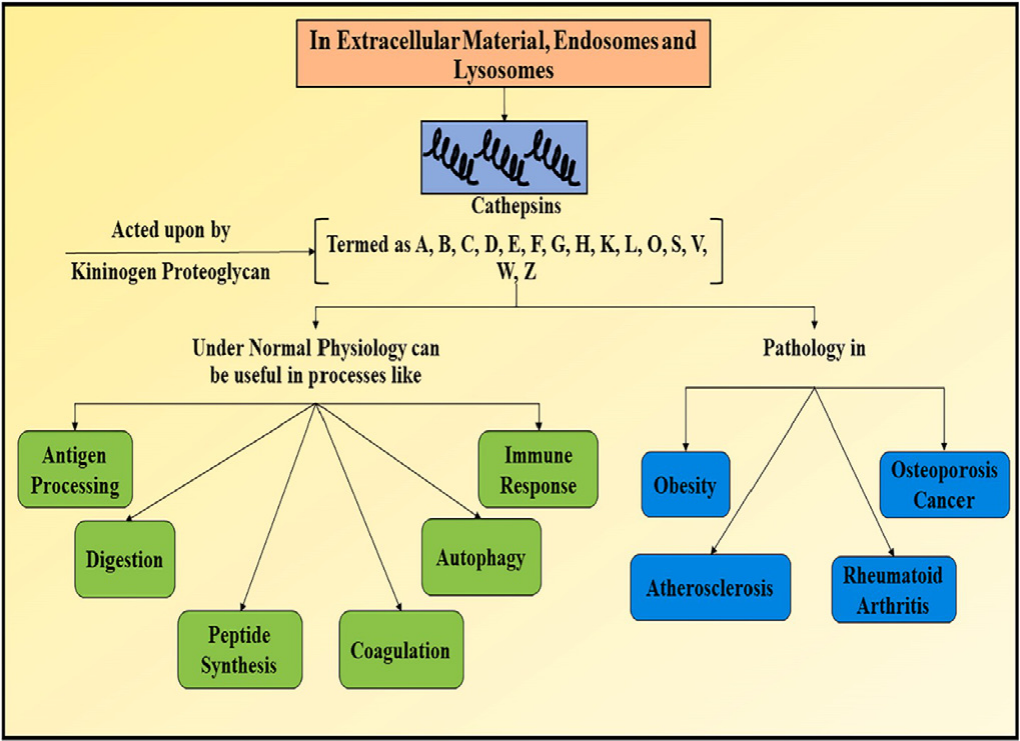
Depicting the role of various cathepsins inside human body under normal physiological conditions and their action in different diseases.

Cathepsins can be defined as the heterogeneous group of proteases which are found inside lysosomes and possess acidic environment. Cathepsin is derived from a greek work Kathepsein which means to digest. Cathepsin family comprises of 15 members, which are divided on the basis of their catalytic activity. The three major groups of their distinction include serine proteases (contains cathepsin A and G), aspartate proteases (cathepsin E and D, and cysteine proteases (includes cathepsin B, C, F, H, K, L, O, S, V, W, and X). Most of the cathepsin (expect E and G), reside inside endosomal compartment. Hence, they are termed as lysosomal cathepsins. These are termed as intracellular active enzymes which are accountable for proteolysis inside acidic environment, where they degrade intracellular as well as extracellular proteins. They also exert some functions under normal conditions which are shown in Fig. 2 (Fig. 2). Cathepsin B is an exopeptidase as well as endopeprtidase, and also acts as peptidyldipeptidase (Conus and Simon, 2008, Sloane et al., 1981, Burleigh et al., 1974). Cathepsins acting as endopeptidases contains cysteine cathepsin K, L, S and V (L2). Particular involvement of cathepsin B is reported in osteoarthritis and rheumatoid arthritis where it causes cartilage destruction. At initial stages amplified concentration and expression of cathepsin is observed and with the advancement in the disease, its expression attenuates due to developed stage of degeneration. Molecular biologics data have also suggested the role of cathepsin B in the cartilage degeneration (Roshy et al., 2003, Guay et al., 2000, Turk et al., 2001).

The activity of cysteine cathepsin is based on their pH as well as their cellular localization. The characterization of cysteine cathepsin reveals that it comprises of cysteine residue at the active site. Their structure is homologous to that of papain (which is a cysteine protease obtained from the papaya fruit) and thus are termed as papain like cysteine protease. Cathepsin which are in combination with parent protease papain are termed as MEROPS which acts as peptidase database. They are expressed inside vertebrates, primitive parasites, plants, and invertebrates. They get transferred inside lysosomes through a specific pathway (mannose-6-phosphate pathway) and can play a potential role in chronic as well as infectious disease. Matured proteolytic active cathepsin gets released after their activation via the removal of N-terminal propeptide, which occurs at low pH of lysosomes (Turk et al., 2001, Rawlings et al., 2007, Lee-Dutra et al.,). The various cathepsins serving their role in rheumatoid arthritis are discussed below in the later sections.

## 3 Cathepsin G and its mechanistic role in rheumatoid arthritis

Cathepsin G also termed as CTSG belongs to the family of serine proteases, which was initially detected inside azurophillic granules of neutrophil leucocytes. They are also detected inside myeloid cells, including primary human monocytic cells, myeloid dendritic cells, B cells, murine microglia and plasmacytoid dendritic cells. CTSG is a 255-amino acid residue protein, on undergoing cleavage it leads to formation of 2 amino acids and 11 amino acids residue at N- and C-terminal side of pro-CTSG. Initially it gets stored inside the primary granules and after being stimulated via immune complexes or through pharmacological agents CTSG gets released to the extracellular space. Cathepsin G exert its numerous functions as it can remove pathogens and can regulate inflammation via attenuating the level of cytokines, chemokines and cell surface receptors. They have become a new target for biomarkers and thus they can serve as the target for auto-immune disease (Miyata et al., 2007, Velvart and Fehr, 1987, Siming et al., 2018). They lead to inflammation via promoting migration and transportation of monocytes, chemokines, neutrophils, and antigen promoting cells (APC). It converts prochemerin into chemerin which acts as novel chemoattractant factor and attracts APCs via the receptors. CTSG causes cleavage of chemokines at N-terminal/C-terminal which leads to activation of formyl peptide receptor as well as CC-type chemokine receptor. The activation of receptor leads to recruitment and attraction of monocytes, neutrophils as well as leukocytes. Cathepsin G leads to the promotion of inflammation via activation of cell surface receptors. The activation of protease activated receptors 4 (PAR4) takes place via cathepsin G. Activation causes secretion, aggregation, interaction between platelets and neutrophils, ultimately causing inflammation and vascular injury. It further activates protease activated receptors 2 (PAR2), which leads to secretion as well as production of interleukin-8, monocyte chemoattractant protein and leads to inflammation (Siming et al., 2018, Nordström et al., 1996, Pettipher et al., 1990). The monocytic stimulation also occurs which via releasing CD23 fragments and leads to production of pro-inflammatory cytokines as well as oxidative burst. It is scientifically proved that CTSG causes the degradation of an immuno-2dominant myelin basic protein (MBP) epitope (MBP85-99) which causes its immuno-pathogenesis in multiple sclerosis. Cathepsin G exert its important role in conversion of pro-insulin into numerous intermediates and fragments, each can lead to T cell activation in type 1 diabetes. Cathepsin G leads to augmented production of antigen specific antibody by activating T cells. They bind to CD8+, CD4+, B cells, and thrombin like receptors which enhances cytotoxicity of natural killer cells (Pettipher et al., 1990, Burster et al., 2010, Huang et al., 2020).

Cathepsin G can alter the cell shapes which brings about an intercellular gap between endothelial and epithelial cells, thereby enhancing their permeability. Possible mechanism of action involves attenuating calcium balance which causes amplified inositol phosphate level and thus activates protein kinase C leading to amplified albumin flux across cell membrane, ultimately causing cleavage of endothelial cadherin (which maintains vascular integrity). Evidences have shown neutrophil surface bound proteases causes cleavage of vascular endothelial cells via recruitment of neutrophils. It causes enhanced permeability of type II epithelial monolayers, further leading to structural changes and intercellular gaps which can be observed through scanning electron microscopy (Wilson et al., 2008, Tkalcevic et al., 2000, McDonnell et al., 1993). Paracellular permeability of intestinal epithelial membrane occurs via PAR4 which enhances leads to phosphorylation of myosin light chain and thus can lead to ulcerative colitis. The degradation of tissue remodelling gets affected via the matrix which occurs as a result of activated matrix degrading metalloproteinases. Cathepsin G activates promatrix metalloproteinase-2 which participates in collagen gel contraction, tumour invasion, angiogenesis and capillary tube regression. CTSG serve its role in the pathogenesis of auto-immune disorder like rheumatoid arthritis. The activity of CTSG in enhanced inside synovial fluids of patients with rheumatoid arthritis. It acts as monocyte chemoattractant and as a result recruits monocytes inside synovial lesions. Cathepsin G can lead to degradation of articular cartilage inside cartilage-pannus junction (Travis et al., 1980, Sambrano et al., 2000). CTSG acts as the antigen of the antibodies like anti-neutrophil cytoplasmic antibodies. It enhances monocyte chemotaxis and acts as crucial antigen for anti-neutrophil cytoplasmic antibodies in case of systemic lupus erythematosus (SLE). In an experimentation, role of cathepsin G in degradation of articular cartilage was observed. For this antibodies and peroxidase-anti-peroxidase staining was used against Cathepsin G and applied to superficial articular cartilage. From the observations it was studied that three out of ten patients suffering from rheumatoid arthritis reported for restricted local deposits of cathepsin G and elastase. The observations also showed that inside Polymorphonuclear leukocytes, enhanced activities of elastase, esterase and cathepsin G is observed. Overall findings concluded that cathepsin G and elastase are involved in termination and breakdown of rheumatoid arthritis cartilage (Starkey and Barrett, 1996, Tamiya et al., 2006, Miyata et al., 2007, Zhao et al., 1998).

## 4 Cathepsin K and its significant role in rheumatoid arthritis

Initially, cathepsin K was extracted as cDNA using osteoclast library of rabbit, which is termed as OC-2. Later on three different group were isolated for human homologues which were termed as cathepsin K, X and O2. Predominantly, cathepsin K is present in abundant amount in skeleton (mainly inside osteoclasts). Systematic analysis has shown the observed role and action of cathepsin K inside human skeleton. They have their central action inside osteoclastic resorption of bone matrix. Cathepsin K is abundantly present inside osteoclast. To lesser extent, it is observed inside human heart, skeletal muscle, ovary, testes, small intestine, lung, and colon. Cathepsin inhibitors like leupeptin was able to inhibit the process of resorption inside the osteoclast cell assays. The cells treated with cysteine protease inhibitor comprises of vacuoles which contained collagen fibrils in undigested form. This indicates that degradation of collagen is initiated via cysteine protease (Salminen-Mankonen et al., 2007, Asagiri et al., 2008, Skoumal et al., 2004, Hummel et al., 1998). Cathepsin K has its major role in cleavage of triple helical collagen inside the helical domains. Mutations inside human cathepsin K gene can lead to pycnodysostosis which is an autosomal osteopetrotic disease, which is characterized via skull deformities, short stature and skeletal abnormalities. Recent studies and data have suggested the role of cathepsin K in the pathogenesis of rheumatoid arthritis. As per the studies and previous literature, augmented expression of cathepsin K is observed in the arthritic cartilage. Along with this, a correlation is also studied in between the cathepsin K mRNA level and the severity of rheumatoid arthritis and osteoarthritis. Enhanced levels of cathepsin K are observed inside the synovial tissues and the joints of patients affected with rheumatoid arthritis. The evidence which states and proves the existing role and action of cathepsin K in targeted pathogenesis of rheumatoid arthritis, are observed from pH measurements (done on deteriorating articular cartilage). The established and confirmed values ranges between 6.2 and 5.5 on affected cartilage surfaces (Hao et al., 2015, Skoumal et al., 2008, Wang et al., 2004, Yasuda et al., 2005). These pH measurements suggest that enzymes activate at the acidic pH where it participates in the cartilage destruction. Cathepsin K also exert its collagenolytic activity which amplifies via chondroitin sulphate molecules (mainly via chondroitin-4-sulphate molecules). These chrondroitin sulphate oligomerize with molecules of cathepsin K and this participates in the matrix degradation (Zaidi et al., 2001, Je et al., 2009, Kramer et al., 2017, Vizovišek et al., 2020, Bali et al., 2001). Cathepsin K exerts potent aggrecan degrading capacity which can particularly potentiate collagenolytic activity towards type I and type II collagen. Enhanced cathepsin K in joint can originate inside joint cavity via multiple cellular origin pathway. Expression of cathepsin K gene is observed inside articular cartilage, chondrocytes, and synovial tissue. Macrophages and synovial fibroblast also express cathepsin K gene. Under normal conditions the level and expression of the gene is low while enhanced expression is observed during arthritis. Giant cells that are formed at the later stages during rheumatoid arthritis exhibits higher concentration of cathepsin K mRNA. Cathepsin K thus principally serves its functioning of digesting the cartilage as well as bone fragments which are shared from the joint surface. The phagocytic cells which express cathepsin K cells are known to represent the blood derived macrophages. Activation of cathepsin K is also observed inside synovial membrane. During inflammatory conditions, the proliferated synovium leads to the formation of aggressive tissue/pannus which invades inside articular structures and destroys them (Garnero et al., 1998, Li et al., 2000, Garnero et al., 1998, Wilson et al., 2009, Li et al., 2002).

In patients with rheumatoid arthritis, it has been observed that cathepsin K gets localized inside the synovial fibroblast, macrophages like synoviocytes as well as in stromal multinucleated giant cells. Under normal circumstances, expression of cathepsin K is restricted towards the fibroblast like cells and during rheumatoid arthritis, cathepsin K positive osteoclast are observed in pannus region where it invades inside bone. The invasion of rheumatoid pannus inside subchondral bone brings synovial fibroblast in nearby proximity to that of the osteoclasts. Along with cathepsin K, enhanced production of osteoclast activating factors including receptor activation of nuclear factor-B ligand (RANKL) causes enhanced resorptive activity and thus contributing towards bone as well as cartilage erosion. Expression and activity of cathepsin K around lymphocytic infiltrate causes facilitated transportation of mononuclear cells via *peri*-vascular interstitial matrix. Amplified expression of cathepsin K is associated with Larsen score which reflects the destruction and radiological changes inside joints. The level of cathepsin K-derived type I collagen telopeptides don’t have a specific correlation with bone degradation and its loss. The above data supports a potential role of cathepsin K in destruction of articular cartilage as well as subchondral bone. The various factors which induce production of cathepsin K inside the synovium comprises of TNF-α and IL-1. Cathepsin K respond towards IL-1 as well as TNF-α and enhances their production. Enhanced production of cathepsin K can lead to the invasion of RA synovial fibroblast at pannus cartilage. Thus, cathepsin K serves its valuable action for the evaluation of joint destruction in patients suffering from rheumatoid arthritis and the potential candidate which can inhibit cathepsin can serve as potential therapy, thereby preventing joint destruction (Wilson et al., 2009, Li et al., 2002, Troen, 2006, Kiviranta et al., 2005).

## 5 Cathepsin S, cathepsin L and its role in rheumatoid arthritis

Cathepsin S serve as potent protease comprising of wide range of substrate including elastin, albumin, and insulin B-chain and hemoglobulin. Out of all these, high elastolytic activity of cathepsin S serve its major role in rheumatoid arthritis. Enzyme elastase serve its potential activity inside extracellular matrix at neutral pH. Cathepsin S is found in dendritic cells, B cells and in macrophages. The higher expression of cathepsin S is observed inside lungs where it can serve its role in lung diseases. Lower transcriptional levels of cathepsin S are ascribed to have their role in osteoarthritis multinucleated giant cells which are responsible for removing synovial bone debris. The above finding may indicate the role of cathepsin S in extracellular matrix degradation. Cathepsin S exert its potent proteoglycan degrading action, thereby serving its role in hydrolysing the aggrecans at acidic as well as neutral pH. It is expressed inside synovial macrophages in patients with rheumatoid arthritis. The secretion of cathepsin S inside cartilage matrix during rheumatoid arthritis can serve as an inflammatory process which can be deleterious. This results in alteration of integrity of the aggrecan type II cartilage and collagen. Cathepsin S is a critical protease required in antigen presentation. On comparison with Cathepsin K, it is observed that cathepsin S acts as weak collagenase and thus the degradation/damage via cathepsin S is lesser as in relation to cathepsin K (Nakagawa et al., 1999, Weitoft et al., 2015, Gupta et al., 2008, Hou et al., 2002, Tejera-Segura et al., 2016).

Cathepsin L belongs to cysteine protease and is abundantly expressed inside thymic cortical cells and are assumed to serve significant role for potential selection of T cells which must recognise non-self-structures. The expression of cathepsin L is triggered via proto-oncogenes (c-Harus and c-fos). It is expressed inside synovial cells where it attaches with bone and cartilage, leading to erosion. Cathepsin L leads to the degradation of proteoglycans as well as collagen compartments (including collagen type IX and XI), thereby promoting bone resorption and contributing towards cartilage and bone destruction. They also interfere with framework system of inflammatory cytokines, mediating the proteoglycan degradation via interleukins (mainly interleukin-1). It facilitates the migration of blood-borne mononuclear cells into synovium, which becomes the most pathogenic factor in conditions like rheumatoid arthritis. Cathepsin L also degrades type IV collagen which acts as major component of basement membrane and also degrades the cartilage components. Cathepsin L exert its major effect in rheumatoid as when compared to osteoarthritis, which is based on the fact that it binds and attaches to hyperplastic synovial lining to the bone. This phenomenon is only observed during rheumatoid arthritis. The degradation of matrix can occur via invasion inside pannus tissue. Thus, from the above outcomes it can be concluded that cathepsin L exert its role in rheumatoid arthritis (Cunnane et al., 1999, Keyszer et al., 1995, Iwata et al., 1997, Taubert et al., 2002).

Another cathepsin which can serve its role in rheumatoid arthritis includes Cathepsin D. Cathepsin D is abundantly produced via chondrocytes and exert its enhanced activity in rheumatoid arthritis. Cathepsin D acts via degrading the central core protein of proteoglycans. This degradation takes place at acidic pH. Literature reports have also suggested that cathepsin D can process pro-active cathepsin L and B into their targeted active forms. Amplified action of cathepsin L and D is observed when both acts simultaneously as this potentiate/augments action of cathepsin L (i.e. enhanced transvascular migration of cells inside the synovium). Cathepsin D serve its potential role via the process of invasion as well its correlation with level of c-myc. From numerous studies it can be demonstrated that altered level of matrix metalloproteinases (such as stromelysin and collagenase) could serve its potential role in rheumatoid arthritis (Keyszer et al., 1995, Taubert et al., 2002).

## 6 Future prospective: Therapeutic approaches on inhibiting the action of cathepsin

From numerous studies it can be reported that pharmaceutical inhibition of cysteine cathepsin can prevent the progression of bone and cartilage destruction in rheumatoid arthritis. The basis for treatment of rheumatoid arthritis includes non-steroidal anti-inflammatory drugs as well as corticosteroids. Proper understanding and advancement in the region of cytokine network can be serve as the base for identification of targeted therapies. Although, targeted therapies can’t cure rheumatoid arthritis but can help in the prevention of the disease progression. The action of cathepsin can be inhibited via implication of cysteine protease inhibitor which are designed as site-directed low molecular weight compounds. The active site of cathepsin proteases comprises of a nucleophillic thiol residue which can be counteracted by using an electrophillic moiety (can also be termed as warhead). This warhead can be placed into a peptide/missile section which gets recognised by substrate binding region of protease. Interaction of missile takes within S-binding sites (specifically at N-terminal or C-terminal of protease substrate). The type of reaction (reversible or irreversible bonding) depends on the type and nature of electrophillic moiety. Irreversible inhibitors that have high potency as well as selectivity are not considered to be suitable drug candidates for treatment of chronic disease like arthritis and osteoporosis. The fact of not using them is that their reactivity will be continuous i.e. it will react over and over again with numerous reactive cysteine protein species leading to toxic/lethal side effects. This can lead to generation of immunogenic haptens which are produced as a result of formation of covalently bound inhibitor-cathepsin adducts (Taubert et al., 2002, Gupta et al., 2008, Brömme and Lecaille, 2009, Esser et al., 1994).

Under physiological conditions, action of cathepsins is regulated via the presence of endogenous inhibitors which are termed as cystatins. The most widely implicated cystatin is cyastatin C which acts extracellularly. It serves as cysteine inhibitor which exert its wider action and is linked with arthritis. It can act via treating the chronic and severe arthritic cartilage lesions which can in turn decrease its progression. Down-regulation of cystatin C can lead to articular bone and cartilage damage. Over-expression as well as enhanced levels of cystatin C have their role in rheumatoid arthritis (Barrett et al., 1984, Cimerman et al., 2000). Cathepsin inhibitors (like peptidyl fluoromethyl ketones) can be responsible for the suppression of inflammation and joint erosion in animal models. The clinical findings and application of synthetic cathepsin inhibitors relies not only on the specificity but also on the compounds ability to reach to the target enzyme/cell via intracellular compartment. The major challenge is the cell targeting of synthetic drugs (Ahmed et al., 1992, Peet et al., 1990, Imperiali and Abeles, 1986). Water soluble cathepsin K inhibitors conjugate exert lysosomotrophic features, although high molecular weight conjugates are less potent. Conjugates exert their action because of their ability to inhibit lysosomal activity of cathepsin (Wang et al., 2002). Over the past decade, a number of studies have been done on cathepsin inhibitors and various patents have been issued in concern with cysteine protease inhibitors comprising barhead (including nitriles, aldehydes, lactams as major groups).

Cathepsin K acts as a major target for pharmacological utilization and this has been tested and initiated via many pharmaceutical companies. Only few companies have achieved the target and are under advanced stages of the development. Primarily the research related to cathepsin K was observed by SmithKline Beecham which later accomplished into GlaxoSmithKline. With passage of time, numerous studies were conducted by Axys Pharmaceuticals (accommodated into Celera Genomics), Aventis, Novartis, Bayer, and Merck. Novartis under the clinical trials reported for the production/development of cathepsin K inhibitor (named as AAE581), which was implied for the treatment of rheumatoid arthritis and osteoporosis (in 2003, http://www.novartis.com). The compound is given via oral route and dosage given is 10–50 mg/day. The reports of phase II demonstrated that it inhibits breakdown of collagen and thus improves bone formation (Kim and Tasker, 2006, Palermo and Joyce, 2008, Rodan and Duong, 2008). Another study conducted by GSK demonstrated about 462,795 (cathepsin inhibitor which can be administered orally) and is used for the treatment of postmenopausal osteoarthritis, rheumatoid arthritis and osteoporosis (http://www.gsk.com). Numerous cathepsin K inhibitors have been synthesized and explored on the basis of their stability studies as well. Along with this their capacity to inhibit bone resorption was also studied. Peptide aldehyde inhibitor, inhibits cathepsin K and further it inhibits osteoclast mediated bone resorption inside human assays and animal assays. This improves bone and cartilage loss as well. The major problem with synthesis of cathepsin K inhibitors are mainly because of the lack of tissue specificity. The drug carrier such be such that it could deliver cathepsin inhibitor to the specific site (which in case of cathepsin K: comprises of synovial fibroblasts of joints). The inhibition can also be brought about by inhibiting the lysosomal compartment which can inhibit cathepsin K secretion as well the inhibition of type II collagen occurs via lysosomal compartment. Inhibiting lysosomal compartment inhibits both these processes. The other targeted therapy and strategy can be via implication of polymer conjugates. Polymer conjugates are low molecular weight inhibitors and acts as water soluble polymer carriers. Polymer conjugates are of greater importance and advantage because of their accumulation inside bone and cartilage targeted moieties such as in hard tissues like arthritic joints. The clinical studies have evaluated the efficacy of two polymer bounded inhibitors (mPEG-I as well as ST-PHPMA-I) which inhibits the activity of cathepsin K inside the synovial fibroblasts (Palermo and Joyce, 2008, Rodan and Duong, 2008, Wang et al., 2004).

Development of Cathepsin S inhibitors are deficient in terms of clinical studies as when compared with that of cathepsin K inhibitors. Various companies are engaged in the development of cathepsin K inhibitors, but till date no advancement/success is achieved. The studies related to cathepsin S inhibitor are only related to that of preclinical evaluation. Cathepsin S serves a role in mediating MHC-II immune response, thus the drug/molecular compound targeted for treating the disease which are auto-immune in nature. Primarily studies were conducted by Medivir UK and Peptimmune (in 2004), which formulated an inhibitor of cathepsin S (MV57471). This served as a potential inhibitor and served its role in treatment of disease like rheumatoid arthritis, organ/graft rejection, multiple sclerosis, and pain. They can also serve their potential role in diseases like lupus and diabetes. Medivir lead to the development of another cathepsin S inhibitor which acts as a reversible inhibitor and serve its role in immune response towards antigens. Aventis and celera genomics are working on the development on cathepsin S inhibitors which can be the lead for treating numerous inflammatory and auto-immune disorders (Vasiljeva et al., 2007, Leung-Toung et al., 2002, Leung-Toung et al., 2006). The drug can be implicated for conditions like chronic obstructive pulmonary disorder, multiple sclerosis, asthma, and rheumatoid arthritis. The various warheads which have their implicated role in rheumatoid arthritis and other inflammatory disorders are tabulated in Table 1, Table 2.

**Table 1:** Description of Drugs Used Against Rheumatoid Arthritis.

**Drug**	**Mechanism of action**	**Side effects**	**References**
**Methotrexate**	Acts via inhibiting production of proinflammatory cytokines Also inhibits the proliferation and induces adenosine release.	Sickness, numerous blood disorders, ulcers, mouth disorders and nausea.	(Valerio et al., 2021, Guo et al., 2021)
**Myocrisin (GSTM), Auranofin**	Acts via inhibiting production of pro-inflammatory cytokines. Forms a complex with cysteine protease which prevents bone resorption and process of antigen presentation	Mouth ulcers, blood disorder, kidney disorders, skin rashes, sore throat.	(xxxx)
**Leflunomide**	Inhibits de-novo pyrimidine synthesis as well inhibiting pro-inflammatory cytokine production	Hair loss, skin rashes, nausea, diarrheoa, skin rashes and headache.	(Singer and Gibofsky, 2011, Behrens et al., 2011)
**Infliximab, Anakinra (TNF-a and IL-1 blocking mAb) Etanarcept (TNF-a receptor antagonist**	It show its actions via down-regulating the cytokine response of T cells.	Confusion, headache, fatique, blood disorder, depression, hot flushes, leukemia, and high risk of infection.	(Chatzantoni and Mouzaki, 2006, Xie et al., 2014, Udalova et al., 2017)
**Sulphasalazine**	Reduces neutrophil adherence towards endothelial cells Inhibits leukocytes recruitment towards inflamed site.	Reduction of neutrophil adherence to endothelial cells and leukocyte recruitment to inflamed sites	(Zhao et al., 2021)
**Cyclosporin A**	Downregulates the level of pro-inflammatory cytokines via inhibiting calciceurin-mediated desphosphorylation of nuclear factor activated cells.	Loss of appetite, nausea,kidney disorder, gingival, blood disorder and overgrowth,	(Bredemeier et al., 2021)
**Azathioprine**	Inhibits immune response via decreasing proliferation of immune cells.	Blood disorder, liver disorder, enhanced risk of cancer, nausea and dizziness.	(Kiboshi et al., 2021)

**Table 2:** Description of warheads that can be employed as cathepsin inhibitors rheumatoid arthritis treatment.

**Chemical Class of Compound**	**Mode of inhibition (Reversible/Irreversible)**	**Reference**
Aldehyde	Binds Reversibly	(Gontijo et al., 2021)
Cyclic ketone	Binds Reversibly	(Marquis et al., 2001)
Cyclic hydrazide	Binds Reversibly	(Wijkmans and Gossen, 2011)
Ketoamide	Binds Reversibly	(Chen et al., 2010)
Aminoethyl amide	Binds Reversibly	(Kim and Tasker, 2006)
Nitrile	Binds Reversibly	(Ward and Thomson, 2002)
Β-lactam 6-substitured oxepenam	Binds in a reversible manner but acts slowly	(Frlan and Gobec, 2006)
Cyanamide	Binds Reversibly	(Falgueyret et al., 2001)
Acrylamide	Binds Reversibly	(Mons et al., 2019)
Pyrazole	Binds Reversibly	(Wiener et al., 2010)
Vinyl sulfone	Binds irreversibly	(Palmer et al., 1995)
Bis-hydrazides	Binds irreversibly	(Hill and Vederas, 1999)
Acyclic ketones	Binds irreversibly	(Marquis et al., 2001)
Diacyl hydroxamate	Binds irreversibly	(Bromme and Demuth, 1994)

## 7 Conclusion

Cathepsin are involved in diseases like rheumatoid arthritis, and inflammatory processes via matrix degradation. They are expressed inside immune system (inside dendritic cells, macrophages, osteoclasts, synovial fibroblasts, chondrocytes, and B cells) which are involved in cartilage as well as bone turnover. Cathepsin K, S and G serve as major target for progression of rheumatoid arthritis via degrading collagen as well as extracellular matrix. The inhibition of cathepsin can lead to prevention of progression of diseases. Preclinical as well as clinical data encourages and supports the data that cathepsin inhibitors can serve as novel disease modifying and mechanism-based drugs. Action of cathepsins is regulated via the presence of endogenous inhibitors which are termed as cystatins. The most widely implicated cystatin is cystatin C which acts extracellularly. It serves as cysteine inhibitor which exert its wider action and is linked with arthritis. It can act via treating the chronic and severe arthritic cartilage lesions which can in turn decrease its progression.

## 8 Funding

This review received no funding from any agency of public, profitable or commercial sector.

## Declaration of Competing Interest

The authors declare that they have no known competing financial interests or personal relationships that could have appeared to influence the work reported in this paper.
